# Children’s Rough-and-Tumble Play in a Supportive Early Childhood Education and Care Environment

**DOI:** 10.3390/ijerph181910469

**Published:** 2021-10-05

**Authors:** Rune Storli

**Affiliations:** Department of Physical Education and Health, Queen Maud University College of Early Childhood Education, 7044 Trondheim, Norway; rus@dmmh.no; Tel.: +47-99745356

**Keywords:** physical active play, rough-and-tumble play, early childhood education, interactional affordances

## Abstract

While a growing body of evidence highlights the benefits of rough-and-tumble play (R&T) in young children, it remains one of the most challenging kinds of play to support in early childhood education and care environment (ECEC) institutions. The present study explores the occurrence and characteristics of R&T in indoor and outdoor environments in a Norwegian sociocultural context where children can freely choose what, where, and with whom to play. The data consist of 100 randomly recorded two-minute videos, which were coded second by second for R&T. Qualitative analysis reveals that in a physically and culturally supportive environment for R&T, children aged 3–5 years perceive indoor spaces that afford physically active play to be more attractive for R&T than outdoor environments. The findings indicate gender differences related to R&T and how girls and boys use the physical environment in different ways. The quantitative findings are discussed within interactional affordances theory and show that children practise perceptual, motor, and social skills to successfully engage in R&T. Enhanced knowledge of children’s skill acquisition in R&T can support practitioners in developing pedagogical skills to facilitate challenging and safe environments for appropriate indoor R&T for both girls and boys.

## 1. Introduction

Young children’s participation in early childhood education and care (ECEC) programs has increased steadily over the last decades. In recent years, enrolment rates for 3- to 5-year-olds in Organisation for Economic Cooperation and Development (OECD) countries were 87.2% on average (http://www.oecd.org/els/family/database.htm, accessed on 13 April 2021). However, an increasing number of studies show that children’s opportunities for free play have decreased over the same period [1].

Play is a ubiquitous activity among young children. Within ECEC, play is a fundamental avenue for meeting children’s development and learning requirements [2,3]. From the children’s perspectives, play is voluntary, free, self-initiated, and self-controlled while also fun, active, spontaneous, unlimited, and natural [4]. Santer, Griffins, and Goodall [5] defined free play as children choosing what they want to do, how they want to do it, and when to stop and try something else. Children’s spontaneous free play is complex and unpredictable in the way that it may simultaneously exhibit multiple forms, types, and stages [6]. Fromberg and Bergen [7] claimed that children demonstrate their power as agents when they bring their own experiences, ideas, perceptions, and creativity into the play situation to develop it in spontaneous and unpredictable ways.

The present study explores what happens when children in ECEC institutions have free access to spaces that afford physically active play, such as rough-and-tumble play.

### 1.1. What Is Rough-and-Tumble Play (R&T)?

Hart and Tannock [8] defined R&T as “verbally and physical cooperative play behaviour involving at least two children, where all participants enjoyably and voluntarily engage in reciprocal role-playing that includes aggressive make-believe themes, actions, and words; yet lacks intent to harm either [child] emotionally or physically”. R&T play has clear dimensions of social play but is also characterised by big body play with and without physical contact with players, sometimes including elements of object play (e.g., sticks and toy guns) [9].

In the same way that children’s spontaneous free play has many expressions/faces, R&T is also complex in its tendency to exhibit multiple characteristics of play simultaneously. Hart and Tannock [8] explained that rough types of play that are difficult to distinctly define could be big body play, risky play, imaginary play (war play), and fantasy play (Super Hero play). As such, the characteristics of R&T extend over a wide range of social interactions, from intimate, gentle, physical contact or tumbling [9,10,11] to vigorous behaviours that would appear to be aggressive were it not for the playful context [12].

While research increasingly focuses on the benefits of R&T in young children, it remains one of the most challenging kinds of play to support in ECEC institutions [6,13,14,15].

### 1.2. Benefits of R&T

There is a growing body of evidence indicating the role of R&T in children’s development and learning, with benefits for physical health and development, social development, and aggression regulation [8,16]. The value of R&T is similar to the value of play, in general, in children’s development and learning. One such function is that R&T enhances children’s social competencies [14], such as affiliation with peers, social signalling, and management and dominance skills within the peer group [17,18]. Pellegrini [19] postulated that R&T may serve a social function in peer groups, especially for boys, by assisting in the establishment and practice of complex social skills, such as bargaining, manipulating, and redefining situations. In R&T, role reversal, self-handicapping, and self-control are considered essential skills to succeed socially [19], as these are all practical skills that require physical and motor control related to another person.

### 1.3. Consideration of Interactional Affordances for R&T in a Norwegian Cultural Context

The theory of affordances [20] is an important framework for examining individuals’ perceptions of the environment surrounding them. It holds that the physical environment in which one lives affords individual actions and behaviours. Rietveld and Kiverstein [21] maintained that an individual adequately engaging in an affordance is often exercising a skill. Heft [22] argued that a distinctive characteristic of affordances is that they are relationally specified. Affordance properties are simultaneously determined by the attributes of the environmental feature of interest and a particular individual. This person–environment relationship is immediate and based on unreflected, functional activity [23,24], which means that one must perceive to move as well as move to perceive. Clark and Uzzell [25] claimed that Gibson appreciated the importance of social and cultural meanings in environmental perception and believed that the richest and most intricate affordances of the environment are provided by other people. Miranda et al. [26] claimed that high-quality play environments provide various affordances related to spaces, equipment, and materials that encourage social interactions and types of play. The space affords an activity if the activity is perceived as doable in the space by the doers. In an intervention study, Sando and Mehus [27] found that an indoor environment supportive of functional play, such as R&T, is positively associated with physical activity.

Kyttä [24] argued that the concept of affordances also has the potential to be extended to comprise emotional, social, and cultural opportunities that the individual perceives in the environment. Social affordances, a subcategory of affordances, refers to opportunities in the environment that promote social relationships and interactions [23]. Clark and Uzzell [25] reported Gibson’s theory that individuals learn about the social affordances of the environment from other people, recognising a role for development and learning via perception. In R&T, perceiving, for example, play signals and then acting upon them is, according to the theory of affordances, how children gain practical experience and develop social skills through play. Rietveld and Kiverstein [21] stated that by acquiring a skill, the individual increasingly becomes an expert at responding adequately and appropriately to the actions a particular situation invites.

Waters [28] argued that less tangible domains of rules and understandings of behaviour should also be considered to fully understand the complexity of children’s actualisation of the affordances of an environment. In an educational setting, the affordances for interactions are nested within and mediated by the interrelated aspect of the broad sociocultural, physical, and specific local contexts of the space and the individuals within it [28]. This condition is particularly illustrated in the context of R&T, which is a type of play pursued by children but often discouraged by adults [8,13,29].

The Norwegian Framework Plan for the Contents and Tasks of Kindergartens [30] emphasises that children should participate in activities in which they can engage in physical activity, play, and social interaction and experience motivation and achievement according to their abilities. In Norway, children’s right to free play, during which they can freely choose what to play, with whom, and where is regarded as an important element of the content. Kindergartens should also help children get to know their bodies and develop an awareness of their own limits and those of others. As such, arguments for R&T as defined by Hart and Tannock [8] are deeply rooted in the Framework Plan for the Contents and Tasks of Kindergartens [30]. A study within a Norwegian ECEC context [13] showed that play fighting and chase games are the most restricted R&T categories by preschool teachers. However, R&T is, in general, significantly less restricted in outdoor environments than in indoor environments. Sandseter, Kleppe, and Sando [1] examined the prevalence of risky play in young children’s indoor and outdoor free play in ECEC institutions in Norway and revealed that risky play was registered in 10.3% of the total data material (1878 randomly recorded two-minute videos). R&T, as one of the eight categories of risky play, constituted 2.7%. In addition, analyses showed that girls spent significantly less mean time in R&T than boys, and significantly more R&T occurred indoors than outdoors. The explanation of the latter finding may be because many of the participating ECEC institutions in this study had available indoor tumbling spaces where the teachers allowed children to engage in R&T. In their concluding remarks, Sandseter et al. [1] highlighted the importance of ECEC institutions facilitating possibilities for physical active play, such as R&T, both indoors and outdoors, even though the activity might look a little risky to the staff.

### 1.4. Aim of the Study

When asked, children prefer to play in environments where they can explore through child-centred activities and experience joy, fear, excitement, and success [31,32,33]. R&T affords these experiences, yet there is a tendency for educators to restrict this type of play due to being unfamiliar with its benefits. In addition, a lack of pedagogical skills to facilitate challenging and safe environments for such play tends to prevent children from participating in this form of play [8,29,34]. Therefore, this study aims to explore the occurrence and characteristics of children’s R&T in indoor and outdoor environments in a Norwegian sociocultural context where they can freely choose what, where, and with whom to play.

## 2. Materials and Methods

This study is part of the Competence for Developing Early Childhood Education and Care (ECEC) Institutions’ Indoor and Outdoor Environments project, funded by the Research Council of Norway and approved by the Norwegian Social Science Data Services. The project lasted from 2017 to 2020 and was conducted in close collaboration with three owners of ECEC institutions in Norway. The data collection involved systematic and randomised video observations of children in indoor and outdoor environments during free play at two data points (T1 and T2), where free play implied that the children could decide what they wanted to do, where they wanted to be, and with whom they wanted to interact.

### 2.1. Participants

In this study, the participating ECEC institutions were selected from facilities operated by three partnering ECEC owners. The owners made at least twice as many ECEC institutions available as were required for the study. An important criterion for selected institutions was having at least 20 children without special needs, aged 3 to 5 years old, who could be recruited as participants. Eight ECEC institutions were chosen based on a strategic decision to include different types of institutions in terms of size, age of the institution, location, and physical environment.

The Norwegian norm for children in the relevant age group is 4 m^2^ per child for indoor environments and 24 m^2^ per child for outdoor environments. All participating institutions followed the norm according to the number of children in the institution. The ECEC institutions’ indoor environments consisted of a mix of spaces dedicated to smaller groups and spaces for the common use of all groups. All spaces had a variety of furniture, materials, and toys designed to support a wide range of play and daily activities for the children and staff. Seven of the eight participating institutions provided indoor spaces with appropriate play equipment (e.g., soft mats; big, soft play materials; blankets; pillows) that afforded opportunities for big body play.

The outdoor physical environment ranged from small (750 m^2^) urban playgrounds to large (13,000 m^2^) natural environments. All outdoor playgrounds had traditional equipment for physically active play, such as swings, slides, and climbing facilities, and followed the norm of at least 24 m^2^ of outdoor space per child.

Once informed consent was obtained from all the children’s parents, five girls and five boys who consented to participate were randomly selected from each institution by manually drawing names from a box. The final sample consisted of 86 children: 80 at T1 (autumn 2017) and 79 at T2 (autumn 2018). The distribution of gender between T1 and T2 was nearly equal, with 51% of the observations involving boys and 49% involving girls. The children’s mean age was 3.8 years (SD = 0.6) at T1 and 4.7 years (SD = 0.6) at T2.

### 2.2. Procedure and Data

All observations were video-recorded and performed in accordance with a systematic and strict protocol that ensured random sampling of observational sequences and identical methods of data collection at each institution. The protocol instructed the data collector to perform each observation by recording child 1 for 2 min, followed by a 6-min break to locate the next child in the play area. Next, child 2 was recorded for 2 min, followed by another 6-min break to locate child 1 for his or her second round of observation, and so forth. The final sample included 950 observations at T1 and 928 observations at T2, with an average of 21.8 (SD = 4.3) observations per child. Thus, 42 observations were missing. Missing observations occurred because children were sick or picked up early from the ECEC institution, while other observations were excluded because the child was hidden from view, preoccupied with the recording equipment, or because a technical or human error occurred. The number of missing video observations is low and does not pose a methodological challenge for the present study.

### 2.3. Coding of R&T, a Subcategory of Risky Play

The data consist of 1878 randomly recorded two-minute videos. In step 1, risky play was coded using the Observer XT 12.5 behaviour coding (Noldus) analysis and management software for observation data [35]. This software allows for second-by-second coding of the videos, so three independent assessors coded the instances and duration of the various types of risky play. For a thorough description of the coding of risky play, see Sandseter, Kleppe, and Sando [1]. The results from step 1 revealed that risky play was registered in 10.3% of the total data material, and 2.7% of the material was coded R&T (n = 100).

### 2.4. Data Analysis

Transcriptions of the observations from the video recordings coded R&T in Sandseter et al. [1] constituted the data material that formed the basis of the analyses. Initially, all observations (n = 100) coded R&T were carefully transcribed according to the aim of the study.

Then, in step 2, theoretical thematic analyses of play characteristics were performed based on the following predetermined categories and characteristics of R&T described by Aldis [12], Pellegrini [19], Pellis et al. [14], and Tannock [11]:

1. Chasing games: two or more children run and chase each other. Role-reversal often observed. Can involve fragmentary dimensions of categories 3 and 4 in between the chasing. Low/some degree of competition between the players.
2. Big body play without contact: large motor physical activity, such as rolling, jumping, falling, and sliding, without physical contact with other players. Play and exploration of affordances in the environment, one or more children together. No competition between players.
3. Big body play with contact: large motor physical tumbling involving two or more children, with or without play equipment. Low degree of competition between the players.
4. Play fighting: boxing, kicking, pushing, and wrestling. Obvious degree of competition and play aggression between players. Often combined with imaginative play (e.g., superhero play).

Although Hart and Tannock [8] maintain that educators should focus on the characteristics and context of play behaviour rather than its given name, each observation was categorised based on the dominant play characteristics of the observation.

Step 3 was to analyse the social interactions that occurred in the data material, which means with whom the observation child communicated and played with.

Step 4 in the analysing process was to explore the occurrences when children were playing (i.e., indoors, outdoors) and describe the characteristics in the play environment that afforded R&T incidents (e.g., play equipment, surfaces).

### 2.5. Ethical Considerations

Research with young children involves special ethical issues [36]. One of these issues is the need to gain informed consent from both the parents and the children (in situ before each observation). It is important to ensure that the children understand that they can withdraw from the project at any time. The researchers were highly conscious to refrain from recording children in sensitive situations, such as toileting and changing clothes. The study was approved by the Data Protection Official for Research in Norway under the premise that the data would not be analysed or published at the centre level due to the relatively low number of children at each institution.

## 3. Findings

As described in the method for obtaining the data (n = 1878), 51% of the observations were of boys, and 49% were of girls. In each observation, one child was followed.

Table 1 shows that of the 100 observations of R&T, the observation child was a girl in 28 instances and a boy in 72 instances. While the girls were generally occupied with big body play with and without physical contact with other children (78.5%), boys tended to be more involved in all types of R&T. The most prominent gender difference is that girls performed nearly twice as much (46.4% versus 23.6%) big body play with contact than boys did. Boys engaged in play fighting approximately three times more (26.4% versus 7.1%) than girls did.

An example of continual indoor play fighting between two boys is as follows (fictive names):

*William and Peter are play fighting in the tumbling space of the department. There are more children in the room, both boys and girls, together with a practitioner. While in a standing position, William pushes Peter into a corner and punches his body several times. Peter hits him back, and the two boys end up wrestling on a mat where another boy is sitting. Although William and Peter are almost laying on him, the boy does not seem to notice them. After a brief session of wrestling, they stand up and start punching and chasing each other until they fall down again, wrestling on the floor, first with William dominating Peter, then the opposite. Suddenly, Peter bounces up and distances slightly from William. The boys stare at each other for a moment before William throws a big pillow at Peter. Once again, William pushes Peter into a corner while punching him. Peter hits him back again, and the two end up wrestling on the floor, with Peter on top. Then, William signals that he is hurt, and Peter momentarily responds by retreating and following William carefully from a distance. “My foot hurts”, William says to the practitioner sitting next to him. After a brief “timeout”, William and Peter make eye contact. A few seconds later, William is chasing and capturing Peter, and they continue wrestling in a standing position.*

This observation illustrates how William and Peter use their bodies to communicate in play fighting and adjust their strength and movements in accordance with their partner. Although they do not speak much, they confirm their play intentions through continuous perception (eye contact, smiles, and laughter). The two shift roles between the chaser and the chased and the dominator and the dominated. When William gets hurt, both seem to realise it is an accident and not a purposeful act. Although there are many children in the tumbling space, the two do not receive any specific attention from the others.

Since the data in Table 1 represent types of R&T, the findings are limited to illustrating the distribution of the R&T categories, not how much time the observation child spent in R&T. However, the more registrations in one category, the more time the observation child likely spent in that category of play. In a study based on the same data but quantitatively analysed, Sandseter et al. [1] found that girls spent significantly less mean time in R&T than boys.

To illuminate the gender differences of participation in R&T, a further explorative analysis of whom the observation child was playing with was carried out. In 20 of 28 R&T situations (71.4%) where the observed child was a girl, she played with another girl or group of girls. If the observation child was a boy, he played with another boy or group of boys in 48 of 72 observations (66.7%). However, in 29 of 100 observations, boys and girls played together. In summary, both girls and boys seem to prefer playing with children of the same gender. Thus, it could be argued that R&T separates girls and boys more than it brings them together.

In situations where R&T takes place in mixed gender groups, girls were more likely to adapt to the play pattern of boys, as described in Table 1, than boys acting similarly to girls. Thus, in general, boys participate more in all categories of R&T, especially play fighting. The following observation exemplifies this observation (fictive names):

*“Bill (the observation child, age 4.10 years) is standing next to a climbing device, watching another boy (Tim) and a girl (Eva) walk away from him. Suddenly, he roars, “Ahrrrrr,” and starts running after them. The two start running as well and split up after a while. Bill chases Eva and shouts, “I want you!” when he is close to her. Eva smiles and lets herself be captured by Bill. While Bill is embracing Eva, Tim and another boy (Neil) come to help release her. Eva escapes and runs away quickly, followed by Bill and Neil. Neil tries to prevent Bill from catching Eva. Bill stops and hits Neil’s upper body several times in a controlled manner while he looks at the camera. Bill continues chasing Eva until he captures her again, and they both fall to the ground (Bill is at the bottom). Tim and another girl free Eva from Bill, and they all run away. Bill stops after a while and sits down on a small hill. The other children tease him with words and gestures in a manner to get him involved in the play again. After a brief break, Bill starts chasing his playmates again. He catches up with Tim, and the two start boxing/wrestling until they fall to the ground. After that, they continue the chasing game”.*

This observation illustrates how boys and girls can play together outdoors. In the transcription protocol, the analysis of play categories is described as “chasing games” and “play fighting”. Although most of the play fighting occurs between boys (hitting, boxing, wrestling), some of the wrestling between Bill and Eva is also coded as play fighting. As Table 1 shows, girls’ involvement in play fighting is rare.

In groups with more than two children playing together, the participants often have different roles according to involvement and dominance. In the present observation, Bill keeps the play going, and he is primarily the child that regulates the excitement of chasing. Since he is the most physically capable child (e.g., running speed and physical strength) of the group, he adjusts his running speed to extend the exhilarated fleeing. After capturing Eva, he also performs self-control in the way he embraces and holds her. When Tim and Neil free Eva, the play appears to be getting rougher. The boys box with each other in a rough yet constrained and controlled way. Bill shows sensitivity to the context of the play by seeking eye contact with the practitioner behind the camera, perhaps to confirm “we are just playing” or to obtain permission to continue. Although this play observation is a dynamic process between all participants, it is Bill who keeps the play continuing.

Table 2 shows that in this study, 38 (38%) observations were recorded outdoors, and 62 (62%) observations were recorded indoors (N = 100). In the outdoor environment, chasing games and play fighting were the most prevalent categories of R&T. In the indoor play environment, big body play with and without contact was most prevalent.

Qualitative descriptive video analyses showed that in the outdoor environment, 23 of 38 observations were recorded in open spaces (e.g., grassy or gravel areas), 8 involved play structures (e.g., climbing equipment, slides), 4 were in nature environments, and 3 were in other places (e.g., sandboxes, play huts).

In the indoor environment, 43 of 62 observations were recorded in the tumbling spaces of the departments, 11 in common spaces/open areas in the departments, 3 in other places (e.g., wardrobe), and 5 in special rooms for physical activity.

According to the theory of affordances, children perceive possibilities for R&T by “doing” in the specific environment; this theory argues that there is a strong relationship between the structure of the environment and the functions of play [22]. Open spaces afford activities, such as running, and soft surfaces and play materials encourage actions, such as jumping, rolling, hitting, throwing, and wrestling. In the present study, seven of the eight participating ECEC institutions offered spaces with such structures indoors.

## 4. Discussion

The main aims of this study were to examine the occurrence and characteristics of R&T in indoor and outdoor environments where children could freely choose where, with whom, and what to play.

The theoretical framework for exploring children’s play behaviour in this study is based on interactional affordances theory as described by Waters [28]. The interactional affordances theory emphasises that children’s actions are nested within and mediated by the interrelated aspects of the broad sociocultural context (e.g., facilitation and support of physically active play), the physical context (i.e., indoor versus outdoor play environment), the specific local context of the space (e.g., tumbling spaces), and the individuals within it (i.e., children and teachers). As such, the findings of this study should be locally and contextually interpreted.

Theoretical thematic analyses of play characteristics show that the preschool children’s R&T in this study spans a wide range of social interactions between the players: chasing games, big body play with and without physical contact between players, and play fighting. Since equal numbers of girls and boys were systematically observed in different indoor and outdoor play environments with supportive and present practitioners, one might expect that the field of promoted action explicitly promoted equal possibilities for R&T between girls and boys. However, Table 1 reveals that boys and girls engage in slightly different play patterns related to the play characteristics as described. While boys are nearly equally involved in all characteristics of R&T, girls are most involved in big body play. Play fighting is the type of play that shows the largest gap between girls and boys.

Previous research has reported that boys are more likely than girls to engage in R&T across cultures [8,37]. In 72 of the 100 observations of R&T in this study, the focus child was a boy. As such, the findings in this study are consistent with previous research. Further analysis of social constellations showed that 66.6% of these play observations were boys playing with boys. This pattern of gender segregation in R&T was even more distinct when the focus child was a girl. In 20 of 28 observations (71.4%), girls played together in dyads or groups. Whether gender segregation behaviour arises from nature or nurture is not a central issue in this study. However, since the children were free to choose where they wanted to play and with whom in an environment that supported R&T, the findings can be illuminated using interactional affordance theory. Clark and Uzzell [25] stated that Gibson recognised a role for learning and development in perception, claiming that people learn about the social affordances of the environment from other people. As such, perceiving play signals and then acting upon them is how children gain practical experience and develop social skills through play. An individual adequately engaging with an affordance is often exercising a skill, according to Rietveld and Kiverstein [21]. In the aforementioned R&T play episode in the mixed gender group (page 6), Bill responded adequately and appropriately to the social affordances in the particular situation and exerted actions and social skills related to the individuals with whom he was playing. When chasing the other children, he adjusted his running speed relative to his playmates’ running skills to increase the duration and unpredictability of the play. He also treated Eva differently from Neil in the way he individually adjusted his physical strength and acted (e.g., boxing with Neil). In the end, when Bill took a break, the other children teased him with words and gestures to get him to start playing again. Bill’s response to teasing from his playmates shows that he perceived the implicit invitation to continue playing, so he started chasing the other children immediately.

In describing strategies for supporting R&T, Hart and Tannock [8] emphasised a designated play space with a large, soft floor area, an uninterrupted area, group size, and supervision as helpful. In addition, these areas should be uninterrupted from nonparticipating peers and free from learning activities. DiPietro [38] claimed that girls tend to be more verbal, while boys tend to be more physical in identical environmental situations. Although affording spaces for R&T seems to meet boys’ interest in physically active play the most, Table 1 shows that many girls might also appreciate such a facilitation.

Storli and Sandseter [34] found in their study of R&T in a Norwegian context that rough-and-tumble play is significantly less restricted by practitioners in outdoor environments than indoor environments. Since seven of the eight ECEC institutions in this study provided the children with free, daily access to spaces with furniture and play materials that afford big body play, one might assume the practitioners’ attitudes and motivation for R&T are quite similar between indoor and outdoor environments. Kyttä [24] called the facilitated play environment the field of promoted action, which regulates which affordances can be actualised as well as the time, place, and manner in which they can be actualised in a socially approved way. Expressed with affordance vocabulary, in this study, children both perceive and realise affordances for R&T to a greater amount indoors than outdoors. Tumbling mats and large, soft construction materials in an uninterrupted space, supervised by supportive practitioners, offer potential affordances for R&T in a more perceivable way for children than outdoor environments do. Although children primarily learn to perceive affordances they have been encouraged to perceive, affordances will always be available for children to discover independently. In the field of free action, as described by Kyttä [24], the quality and quantity of children’s independently actualised affordances vary by context according to the child’s perceptual, motor, and social skills. In addition, personality traits, personal preferences, individual skills, social affordances, and gender differences influence the field of free action.

As described in the introduction, the Norwegian Framework Plan for Contexts and Tasks of Kindergartens [30] offers a broad definition and understanding of play that includes R&T as defined in this study. However, other studies have shown the tendency for educators to prohibit this type of play due to being unfamiliar with its benefits or lacking the knowledge and skills to pedagogically facilitate R&T learning processes [8,29]. Hart and Tannock [8] argued that because women comprise the vast majority of early childhood and primary educators worldwide, early childhood curricula, environments, and pedagogy are representative of a female perspective, with a majority consensus that all rough behaviour, including R&T, is inappropriate in ECEC settings.

In this study, all participating institutions followed the Norwegian norm of 4 m^2^ of indoor space and 24 m^2^ of outdoor space per child from 3 years of age. In all institutions except one, the children had free, daily access to open and grassy areas outdoors. Indoors, all institutions provided spaces or special rooms for physically active play during at least one of the two observation periods. More specifically, in the second data collection period, seven of the eight institutions provided the children with free access to tumbling spaces that were directly connected to the department where the children normally stayed. This availability of indoor spaces may explain why more R&T incidents occurred indoors than outdoors in Table 2, as the tumbling spaces indoors not only provided spaces for physically active play, similar to the outdoors, but also materials (e.g., soft mats, large construction materials, and big pillows) that afforded activities, such as big body play with and without physical contact.

A functional taxonomy of children’s outdoor environments [22] indicated that open spaces offer children possibilities to engage in activities, such as walking, running, cycling, skating, and chasing games. Heft [22] acknowledged that this taxonomy represents a limited view of the functional possibilities of a particular space, and corroboration by empirical evidence is needed.

The play observation described in the findings (page 7) exemplifies how chasing games are a natural part of R&T behaviour outdoors. Table 2 shows that chasing games take place more regularly outdoors (14 observations) than indoors (3 observations). While outdoor spaces are open and normally designed for physically active play, running is often constrained indoors with rules and physical obstacles, such as doors and furniture. According to Kyttä [24], the actualisation of affordances can be limited through the design of objects and spaces so that users cannot actualise the potential affordances. For example, special rooms for physical activity that one would expect to afford possibilities for R&T were often situated apart from the department and had to be booked in advance. They were, as such, unavailable for children’s free and self-initiated play as part of the daily routine. How buildings are designed and how rooms are organised with furniture and play materials affect children’s possibilities to make independent choices for free action [28]. In this study, the children were free to choose what to play, but constraints within the buildings of positioning of special rooms for physical activity did not promote affordances for R&T specifically. However, since seven of the eight ECEC settings in this study promoted free access to tumbling spaces within the department where the children normally stayed, this setup suggests that the field of promoted interaction represented by the practitioners’ motivation for R&T was generally supportive.

## 5. Conclusions

The present study explored what would happen when children in ECEC institutions had free access to spaces that afforded physically active play, such as R&T. The findings show that children aged 3–5 years perceive indoor spaces that afford physically active play to be more attractive for R&T than outdoor environments. In a physically and culturally supportive environment for R&T, girls and boys utilise the physical environment in different ways. Boys participated more than girls in all categories of R&T, especially play fighting. Girls seem to be attracted to less physically demanding (competitive) categories of R&T, such as big body play with and without physical contact with others. Most (2/3) R&T activities in this study occurred in gender-separated play groups, although the children often played simultaneously within the same space. The quantitative findings in this study are discussed with interactional affordances theory and show that children practise perceptual, motor, and social skills to successfully engage in R&T.

## 6. Implications

This study shows that providing open indoor spaces with rich access to big and soft play materials seems to inspire boys to a greater extent than girls to engage in R&T. However, since participation in R&T is closely connected to children’s social skill development in the literature, ECEC institutions should provide safe but challenging environments that afford R&T for both boys and girls. Enhanced knowledge of children’s skill acquisition in R&T can support practitioners in developing pedagogical skills to facilitate challenging and safe environments for appropriate indoor R&T in ECEC institutions.

## 7. Limitations

The theoretical framework used to explore children’s play behaviour in this study is based on interactional affordances theory as described by Waters [28]. This theory emphasises that children’s actions are nested within and mediated by the interrelated aspects of the broad sociocultural context (e.g., facilitation and support of physically active play), the physical context (i.e., indoor versus outdoor play environment) and the specific local context of the space (e.g., tumbling spaces indoors), and the individuals within it (i.e., children and teachers). As such, the findings of this study should be interpreted locally and contextually. However, the Norwegian context is suitable for exploring R&T and its natural accordance in ECEC institutions due to the emphasis on free play.

The reliability of coding risky play, which constitutes the data for this study, was established by three independent assessors. The author of this article carried out qualitative analyses (transcription and coding of R&T observations). As mentioned in the Introduction, children’s spontaneous free play is complex and unpredictable in that it may simultaneously exhibit multiple forms, types, and stages [6]. Therefore, another assessor could interpret and code the play observations differently and heighten the accuracy of the measure. Nevertheless, developing robust categories with descriptive characteristics may have strengthened the reliability.

The study is part of a competence project whose main objective is, in close and mutual collaboration with the ECEC practice sector, to develop new knowledge and to test new research tools that will result in a higher competence in planning, designing, and developing ECEC institutions’ physical (indoor and outdoor) environments. This context may have affected the participating practitioners’ attitudes and practices in facilitating supportive environments for free, physically active play, such as R&T. Another potential limitation is that the participating children may act differently when they know they are being filmed.

## Figures and Tables

**Table 1:** Distribution of categories of children’s R&T related to the gender of the observation child and social playgroups.

Subjects	Categories of R&T				
Observation Child:	Chasing Games	Big Body Play without Contact	Big Body Play with Contact	Play Fighting	Total
Girl	4 14.3%	9 32.1%	13 46.4%	2 7.1%	28 100%
Boy	13 18.1%	23 31.9%	17 23.6%	19 26.4%	72 100%
Social play groups in R&T					
Girl play groups	1 5%	6 30%	12 60%	1 5%	20 100%
Boy play groups	9 18.6%	17 35.4%	8 16.7%	14 29.3%	48 100%
Mixed play groups	6 20.7%	9 31.0%	8 27.6%	6 20.7%	29 100%
Alone		3 100%			3 100%

**Table 2:** The environments in which R&T occur.

Environment	Categories of R&T				
	Chasing Games	Big Body Play without Contact	Big Body Play with Contact	Play Fighting	Total
Outdoors	14 (35.1%)	5 (13.5%)	8 (21.6%)	11 (29.8%)	38 100%
Indoors	3 (5.2%)	28 (46.6%)	21 (31.0%)	10 (17.2%)	62 100%

## Data Availability

The video material/data in this study are unavailable for individuals others than the researchers involved in this project due to the risk of identifying research participants and the Data Protection Official for Research in Norway’s rules for protection of privacy. The Data Protection Official for Research in Norway has also decided that the data must be deleted by June 2022.

## References

[1] Sandseter E.B.H., Kleppe R., Sando O.J. (2021). The prevalence of risky play in young children’s indoor and outdoor free play. Early Child. Educ. J..

[2] Broström S. (2017). A dynamic learning concept in early years’ education: A possible way to prevent schoolification. Int. J. Early Years Educ..

[3] Garner B.P., Bergen D., Fromberg D.P., Bergen D. (2006). Play development from birth to age four. Play from Birth to Twelve: Contexts, Perspectives, and Meanings.

[4] Wiltz N.W., Fein G.G., Fromberg D.P., Bergen D. (2006). Play as children see it. Play from Birth to Twelve Contexts, Perspectives, and Meanings.

[5] Santer J., Griffiths C., Goodall D. (2007). Free Play in Early Childhood. A Literature Review.

[6] Hewes J. (2014). Seeking Balance in Motion: The Role of Spontaneous Free Play in Promoting Social and Emotional Health in Early Childhood Care and Education. Children.

[7] Fromberg D.P., Bergen D., Fromberg D.P., Bergen D. (2006). Introduction. Play from Birth to Twelve Contexts, Perspectives, and Meanings.

[8] Hart J.L., Tannock M.T., Smith P.K.R.J.L. (2018). Rough play: Past, present, and potential. The Cambridge Handbook of Play: Developmental and Disciplinary Perspectives.

[9] Reed T., Brown M. (2000). The expression of care in the rough and tumble play of boys. J. Res. Child. Educ..

[10] Storli R. (2013). Characteristics of indoor rough-and-tumble play (R&T) with physical contact between players in preschool. Nord. Barnehageforskning.

[11] Tannock M.T. (2011). Observing young children’s rough-and-tumble play. Australas. J. Early Child..

[12] Aldis O. (1975). Play Fighting.

[13] Storli R., Sandseter E.B.H. (2015). Preschool teachers’ perceptions of children’s rough-and-tumble play (R&T) in indoor and outdoor environments. Early Child Dev. Care.

[14] Pellis S.M., Pellis V.C., Reinhart C.J., Wortman C., Plotsky P., Schechter D. (2010). The evolution of social play. Formative Experiences: The Interaction of Caregiving, Culture, and Developmental Psychobiology.

[15] Flanders J.L., Leo V., Paquette D., Pihl R.O., Séguin J.R. (2009). Rough-and-tumble play and the regulation of aggression: An observational study of father-child play dyads. Aggress. Behav..

[16] Dodge K.A., Coie J.D., Pettit G.S., Price J.M. (1990). Peer status and aggression in boys’ groups: Developmental and contextual analyses. Child Dev..

[17] Humphreys A.P., Smith P.K., Smith P.K. (1984). Rough-and-tumble in preschool and playground In Play in Animals and Humans.

[18] Pellegrini A.D., Smith P.K. (1998). Physical Activity Play: The Nature and Function of a Neglected Aspect of Play. Child Dev..

[19] Pellegrini A.D., Smith P.K., Hart C.H. (2002). Rough-and-tumble play from childhood through adolescence: Development and possible functions. Handbook of Childhood Social Development.

[20] Gibson J.J. (1979). The Ecological Approach to Visual Perception.

[21] Rietveld E., Kiverstein J. (2014). A Rich Landscape of Affordances. Ecol. Psychol..

[22] Heft H. (1988). Affordances of children’s environments: A functional approach to environmental description. Child. Environ. Q..

[23] Rietveld E., De Haan S., Denys D. (2013). Social affordances in context: What is it that we are bodily responsive to. Behav. Brain Sci..

[24] Kyttä M. (2004). The extent of children’s independent mobility and the number of actualized affordances as criteria for child-friendly environments. J. Environ. Psychol..

[25] Clark C., Uzzell D.L., Spencer C., Blades M. (2006). The socio-environmetal affordances of adolescents’ environments. Children and Their Environments: Learning, Using and Designing Spaces.

[26] Miranda N., Larrea I., Muela A., Barandiaran A. (2017). Preschool Children’s Social Play and Involvement in the Outdoor Environment. Early Educ. Dev..

[27] Sando O.J., Mehus I. (2019). Supportive indoor environments for functional play in ECEC institutions: A strategy for promoting well-being and physical activity?. Early Child Dev. Care.

[28] Waters J., Waller T., Ärlemalm-Hagsèr E., Sandseter E.B.H., Lee-Hammond L., Lekies K., Wyver S. (2017). Affordance theory in outdoor play. The SAGE Handbook of Outdoor Play and Learning.

[29] Logue M.E., Shelton-Harvey H. (2010). Preschool Teachers’ Views of Active Play. J. Res. Child. Educ..

[30] NMER Framework Plan for the Kindergartens. https://www.udir.no/globalassets/filer/barnehage/rammeplan/framework-plan-for-kindergartens2-2017.pdf.

[31] Ernst J. (2018). Exploring Young Children’s and Parents’ Preferences for Outdoor Play Settings and Affinity toward Nature. Int. J. Early Child. Environ. Educ..

[32] Hyvönena P., Kronqvistb E.-L., Järveläa S., Määttäa E., Mykkänena A., Kurkia K. (2014). Interactive and Child-Centred Research Methods for Investigating Efficacious Agency of Children. Varhaiskasvatuksen Tiedelehti J. Early Child. Educ. Res..

[33] Sandseter E.B.H. (2010). Scaryfunny: A Qualitative Study of Risky Play among Preschool Children.

[34] Storli R., Hansen Sandseter E.B. (2017). Gender matters: Male and female ECEC practitioners’ perceptions and practices regarding children’s rough-and-tumble play (R&T). Eur. Early Child. Educ. Res. J..

[35] Zimmerman P.H., Bolhuis J.E., Willemsen A., Meyer E.S., Noldus L.P. (2009). The Observer XT: A tool for the integration and synchronization of multimodal signals. Behav. Res. Methods.

[36] Fine G.A., Sandstrom K. (1988). Researchers and kids. Knowing Children: Participant Observation with Minors.

[37] Fry D.P., Pellegrini A., Smith P.K. (2005). Rough-and-Tumble Social Play in Humans. The Nature of Play. Great Apes and Humans.

[38] DiPietro J.A. (1981). Rough and tumble play: A function of gender. Dev. Psychol..

